# Maltoma of Thyroid: A Rare Thyroid Tumour

**DOI:** 10.1155/2013/740241

**Published:** 2013-02-11

**Authors:** Navisha Latheef, Vijendra Shenoy, M. Panduranga Kamath, Mahesh Chandra Hegde, A. Raghavendra Rao

**Affiliations:** ^1^Department of E.N.T-Head and Neck Surgery, Kasturba Medical College, Manipal University, Mangalore 575001, India; ^2^Department of Otolaryngology, Kasturba Medical College Hospital, Manipal University, Attavar, Mangalore 575001, India

## Abstract

*Introduction*. Primary thyroid lymphomas constitute up to 5% of all thyroid malignancies and can be divided into non-Hodgkin's lymphomas (NHLs) of B- and T-cell types, as well as Hodgkin's lymphomas. Mucosa-associated lymphoid tissue (MALT) lymphomas are a relatively recently recognized subset of B-cell NHLs, and they are listed as extranodal marginal zone lymphomas according to the revised European-American lymphoma classification. *Case Report*. We report an uncommon case of a 44-year-old man, who noted a painless, growing mass on right side of his neck of the three-month duration. Thyroid profile was within normal limits. FNAC showed lymphocytic thyroiditis. The patient underwent a right hemithyroidectomy. The histologic examination and the immunohistochemistry showed an extra nodal marginal B-cell type maltoma (malt lymphoma). CHOP chemotherapy with rituximab was given. The clinical course has been favourable in the first year of followup, with no evidence of local or systemic recurrence of the disease. *Discussion*. Marginal zone lymphoma encompasses a heterogeneous group of B-cell tumours that variously arise within the lymph nodes, spleen, or extranodal tissues. A case of maltoma of thyroid is presented for its rarity and diagnostic dilemmas. *Conclusion*. Maltomas are slow-growing lymphomas. The optimal treatment and followup of patients with thyroid maltomas remain controversial at present.

## 1. Introduction

Extranodal marginal zone B-cell lymphoma mucosa-associated lymphoid tissue (MALT type) frequently occurs in the mucosa of the gastrointestinal tract. However, they may also occur in lungs, salivary glands, skin, and other sites, including the thyroid [1]. Primary involvement of the thyroid is rare, usually arising in the setting of lymphocytic thyroiditis. In the thyroid, a gland normally devoid of lymphocytic tissue, chronic autoimmune thyroiditis (Hashimoto's disease) has been associated with an increased risk of lymphoma, including the MALT type. The coexistence of reactive and neoplastic processes in the thyroid may cause a significant difficulty in diagnosing maltoma using cytology or histology. This has led to the use of immunohistochemistry, flow cytometry, and molecular techniques (Southern blotting, PCR) to confirm or exclude the diagnosis. Clinically, they are characterized by an indolent course and better prognosis than non-MALT lymphomas [1].

## 2. Case Report

**Figure 1:**
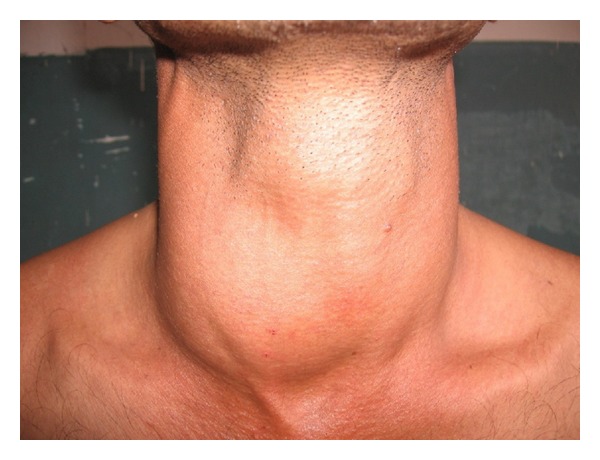
The patient presented diffuse smooth swelling of the right lobe of the thyroid.

A 44-year-old male presented to our outpatient clinic with history of neck swelling on the right side of a three-month duration. It was gradually progressive with no associated pain, weight loss, or pressure symptoms. There was no significant family history. His general physical examination appeared normal without any obvious deformities or abnormalities. Local examination revealed diffuse smooth swelling of the right lobe of the thyroid which was firm in consistency (Figure 1). No features of hypo- or hyperthyroidism were present. There was no retrosternal extension. All his other systems seemed to be normal clinically.

Ultrasound revealed a diffuse enlargement of the right lobe with no calcification. Fine-needle aspiration cytology was suggestive of lymphocytic thyroiditis. Thyroid function tests were normal. All the haematological and biochemical investigations were within the normal range.

**Figure 2:**
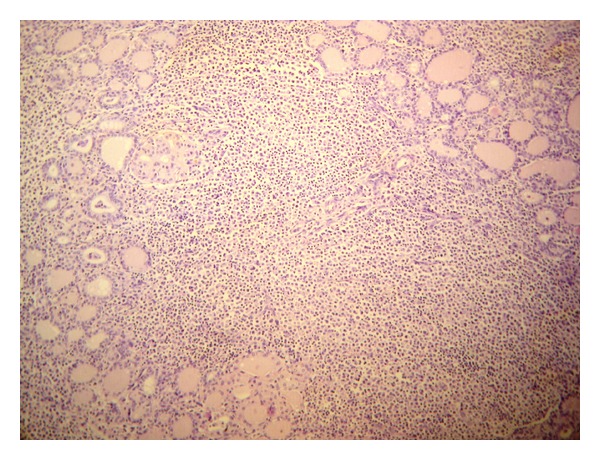
Histopathology slide showing atypical lymphoid cells, which originate within the marginal zone of the lymphoid follicles.

**Figure 3:**
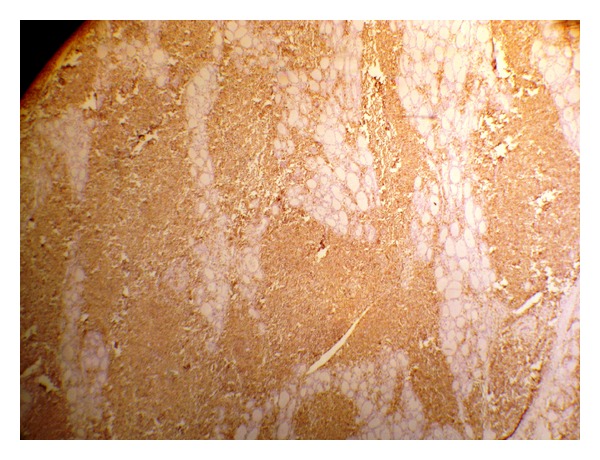
Immunohistochemistry slide.

The patient underwent right hemithyroidectomy. Gross specimen appeared enlarged with no distinct nodules. Histopathological examination of the specimen was suggestive of extranodal marginal zone B-cell lymphoma (Figure 2). Immunohistochemistry showed CD45 positivity in all atypical lymphocytes and centrocyte-like cells (Figure 3). CD3 was positive in reactive T lymphocytes seen focally. CD20 was strongly positive in all the neoplastic B-cells colonizing the thyroid follicles and admixed with sheets of plasmacytoid cells as clusters. Bcl-2 was positive in cells within lymphoid follicles and plasmacytoid cells.

After surgery, whole-body computerized tomographic scan was done. It did not document any pathological finding. A final diagnosis of extranodal marginal B-cell lymphoma (MALT lymphoma) was made. The patient underwent R-CHOP 6-day-cycle chemotherapy and radiotherapy, rituximab 375 mg/m^2^, cyclophosphamide 800 mg/m^2^, vincristine 1.4 mg/m^2^, and Adriamycin 50 mg/m^2^ on day 1. This was followed by prednisolone 100 mg/d from day 2 to day 6. The patient has been symptom-free for more than a year.

## 3. Discussion

Marginal zone lymphoma encompasses a heterogeneous group of B-cell tumours that variously arise within the lymph nodes, spleen, or extranodal tissues. Because these tumours were initially recognised at mucosal sites, they have been referred to as tumours of mucosa-associated lymphoid tissues (or maltomas). In most cases, the predominant cell type resembles normal marginal zone B-cells. Maltomas usually arise within tissues involved by chronic inflammatory disorders of autoimmune or infectious aetiology. Examples are maltoma of the stomach in helicobacter gastritis and maltoma of thyroid arising in Hashimoto's thyroiditis. The appearance of extranodal marginal zone lymphomas in chronically inflamed tissues has led to the suggestion that they lie on a continuum between reactive lymphoid hyperplasia and full-blown B-cell lymphoma. With the acquisition of mutations and chromosomal aberrations, a monoclonal B-cell neoplasm arises on a background of reactive lymphoid hyperplasia [2].

The thyroid gland contains no native lymphoid tissue. Intrathyroid lymphoid tissue is accrued in various pathological conditions, but more evidently in the course of autoimmune thyroid disease, notably chronic autoimmune thyroiditis (Hashimoto's thyroiditis). Histologically, this acquired lymphoid tissue can evolve to lymphoma, including the MALT type [3].

Since the introduction of the concept of MALT-type lymphomas by Isaacson and Wright in 1983, various extranodal locations (including thyroid) have been described. Histologically, thyroid maltomas are characterized by the presence of atypical lymphoid cells, which originate within the marginal zone of the lymphoid follicles and can extend into the interfollicular space and/or into the germinal centres (follicular colonization) [4, 5]. In our patient, FNAC showed groups and scattered round cells with eosinophilic deep stained nucleus with mild anisocytosis with background showing lymphocytes and plasma cells. The picture was suggestive of lymphocytic thyroiditis with follicular neoplasm.

MALT lymphomas express B-cell-associated antigens (CD20, CD22, and CD79a) and are negative for CD5, CD10, and CD3 [6–8]. Immunohistochemistry in our patient showed CD45 positivity in all atypical lymphocytes and centrocyte-like cells. CD3 was positive in reactive T lymphocytes seen focally. CD20 was strongly positive in all the neoplastic B-cells colonizing the thyroid follicles and admixed with sheets of plasmacytoid cells as clusters. Bcl-2 was positive in cells within lymphoid follicles and plasmacytoid cells.

Non-Hodgkin's lymphoma (NHL) may involve the thyroid gland as part of systemic lymphoma or may arise primarily in the thyroid. The most common histological subtype is diffuse large-cell lymphoma. About 3% of NHLs are thyroid lymphomas. The incidence of malignant lymphoma in thyroid is 4%. Women are affected more often than men. The ratio of female : male is 4–8 : 1. The peak incidence is usually in the 7th decade. Patients usually present with a fast-growing mass in the thyroid area of the neck [2, 9].

Maltomas are slow-growing lymphomas. Most of patients remain asymptomatic because the disease remains localized for a long period of time. Usually the diagnosis is confirmed only after histopathology and immunohistochemistry. Surgery is usually undertaken for pressure symptoms or when the diagnosis is in doubt as was the case in our patient where fine-needle aspiration cytology was not conclusive. The optimal treatment and followup of patients with thyroid maltomas remain controversial at present. The usual treatment is radiotherapy for localized disease and chemotherapy for disseminated disease, and recurrence can be treated with either radiotherapy or a combination of radiotherapy and chemotherapy. Retrospective reports suggest indolent behaviour and an excellent clinical prognosis for this subset of thyroid lymphomas [1, 3].

Our patient was treated with right hemithyroidectomy followed by 6 cycles of R-CHOP and radiotherapy, rituximab 375 mg/m^2^, cyclophosphamide 800 mg/m^2^, vincristine 1.4 mg/m^2^, and Adriamycin 50 mg/m^2^ on day 1. This was followed by prednisolone 100 mg/d from day 2 to day 6. The patient has been symptom-free for more than a year.

## 4. Conclusion

Maltomas are slow-growing lymphomas. The optimal treatment and followup of patients with thyroid maltomas remain controversial at present.

## 5. Summary

1. Mucosa-associated lymphoid tissue (MALT) lymphomas are a relatively recently recognized subset of B-cell NHLs.
2. MALT lymphomas are thought to develop from acquired lymphocytic tissue during the course of a chronic inflammatory or an autoimmune process.
3. This case demonstrates that cytology or histology may not distinguish between reactive or low-grade lymphomatous thyroid processes. The use of immunohistochemistry was essential to prove clonality and the presence of lymphoma.
4. Clinically, they are characterized by an indolent course and better prognosis than non-MALT lymphomas.
